# A Genomic Blueprint of Flax Fungal Parasite *Fusarium oxysporum* f. sp. *lini*

**DOI:** 10.3390/ijms22052665

**Published:** 2021-03-06

**Authors:** Anastasia Samsonova, Alexander Kanapin, Michael Bankin, Anton Logachev, Maria Gretsova, Tatyana Rozhmina, Maria Samsonova

**Affiliations:** 1 Mathematical Biology & Bioinformatics Laboratory, Peter the Great Saint Petersburg Polytechnic University, 195251 St. Petersburg, Russia; a.a.samsonova@gmail.com (A.S.); a.kanapin@gmail.com (A.K.); mikle.p.bankin@gmail.com (M.B.); loga4ov@gmail.com (A.L.); 2 Institute of Translational Biomedicine, Saint Petersburg State University, 199034 Saint Petersburg, Russia; mary_grecova@mail.ru; 3 Laboratory of Breeding Technologies, Federal Research Center for Bast Fiber Crops, 172002 Torzhok, Russia; tatyana_rozhmina@mail.ru

**Keywords:** *Fusarium oxysporum* f. sp. *lini*, flax, genome architecture, genome evolution, transposable elements, comparative genomics

## Abstract

Fusarium wilt of flax is an aggressive disease caused by the soil-borne fungal pathogen *Fusarium oxysporum* f. sp. *lini*. It is a challenging pathogen presenting a constant threat to flax production industry worldwide. Previously, we reported chromosome-level assemblies of 5 highly pathogenic *F. oxysporum* f. sp. *lini* strains. We sought to characterize the genomic architecture of the fungus and outline evolutionary mechanisms shaping the pathogen genome. Here, we reveal the complex multi-compartmentalized genome organization and uncover its diverse evolutionary dynamics, which boosts genetic diversity and facilitates host adaptation. In addition, our results suggest that host of functions implicated in the life cycle of mobile genetic elements are main contributors to dissimilarity between proteomes of different *Fusaria*. Finally, our experiments demonstrate that mobile genetics elements are expressed in planta upon infection, alluding to their role in pathogenicity. On the whole, these results pave the way for further in-depth studies of evolutionary forces shaping the host–pathogen interaction.

## 1. Introduction

*Fusarium oxysporum* species complex (FOSC) is a group of soil-borne pathogens causing economically important blights, crown or root rots, and vascular wilts. Although the fungus attacks a wide range of hosts, individual strains exhibit surprising host selectivity towards one species. Strains with the same host range are classified into one *forma specialis* (f. sp.) [1].

Genomics have fueled the studies of *F. oxysporum* ff. spp. genome architecture, revealing remarkable similarities in its organization and sparking an on-going debate on genomic composition and evolvability. The current, prevalent hypothesis suggests a dual compartmentalization of FOSC genomes, dividing them into core and accessory (e.g., lineage-specific (LS)) parts that differ in gene density, composition, and evolutionary dynamics. The core compartment is evolutionary stable both between and within FOSC strains, whereas the variable part is linage-specific and is allegedly solely responsible for host adaptation. The variable compartment features sparse gene density, high transposon abundance, specific repertoire of protein-coding genes. Additionally, in contrast to the core, it carries a large number of variants and exhibits rapid evolutionary dynamics [2,3,4]. 

These observations regarding genome architecture gave momentum to the concept of a “two-speed genome”, as a driver of adaptive evolution [5,6,7]. A horizontal transfer of lineage-specific chromosomes between strains, both pathogenic and non-pathogenic, is feasible and may prove instrumental in host switches, as evidentiated by experiments with pathogens infecting tomatoes and cucurbits [2,8,9,10]. Furthermore, the two-speed model finds an indirect support in polyphyletic host preference of FOSC strains [4] and in grouping of pathogenic strains with non-pathogenic strains in population genomics studies [11,12,13]. 

Over a past decade, however, the two-speed genome model faced criticism as over-simplistic and loosely-defined [7]. Studies of *F.oxysporim* f. sp. *cepae* and *F.oxysporim* f. sp. *lycopersici* (*Fol*) have clearly demonstrated that three chromosomes attributed to the core compartment of the genome, are more divergent, express a greater number of genes encoding secreted proteins and cell wall degrading enzymes, i.e., genes associated with cell defense and virulence [4,14]. These and other observations challenge the original two-speed hypotheses, paving the way for more sophisticated models based on the notion of the multi-compartmentalized genome reflective of a constant “arms race” between host and parasite. 

Plant cell wall being the main frontline of host defense against pathogen is attacked by a wide range of pathogen-secreted enzymes (CAZYmes, carbohydrate-active enzymes), namely pectinases, cellulases, and hemicellulases [2]. The host fights back with the antifungal compounds, such as phytoalexins, small antifungal peptides, reactive oxygen species, and plant-resistant proteins [15]. The latter respond to fungal effectors, also known as avirulence factors (*Avr*) [16,17]. Perhaps the most well studied among *Avr*s are secreted in xylem (SIX) proteins encompassing 14 distinct families of small cysteine-rich secreted molecules [2]. The combinatorics of SIX genes repertoire is complex and elusive, as their composition is a result of host adaptation or host evasion and is purposely tuned to maximize infection potential [16,18,19]. 

*F. oxysporum* f. sp. *lini* is a cause of a wilt disease in *Linum usitatissimum* (flax), limiting the crop cultivation in almost all flax and linseed growing countries. Recently, we reported genome assemblies of five highly pathogenic *Fusarium oxysporum* f. sp. *lini* (FOLINI) strains: the reference hybrid assembly of monoisolate 39 (MI39) sequenced with SMRT technology and Illumina-based assemblies of strains F329, F324, F282, F287 [20]. Syntenic analyses of MI39 assembly and the reference Fol genome facilitated provisional attribution of FOLINI chromosomes into two genomic compartments. Specifically, chromosomes 1–11 and 16–19 were attributed to core part, whereas chromosomes 12–15 and 20 were classified into accessory or variable compartment. 

The study provides a detailed annotation of *Fusarium oxysporum* f. sp. *lini* genome architecture and presents its comparison with genomes of other FOSC strains, thus putting the flax pathogen into the global context of fungal plant diseases.

## 2. Results

### 2.1. Organization of MI39 Reference Genome

**Figure 1:**
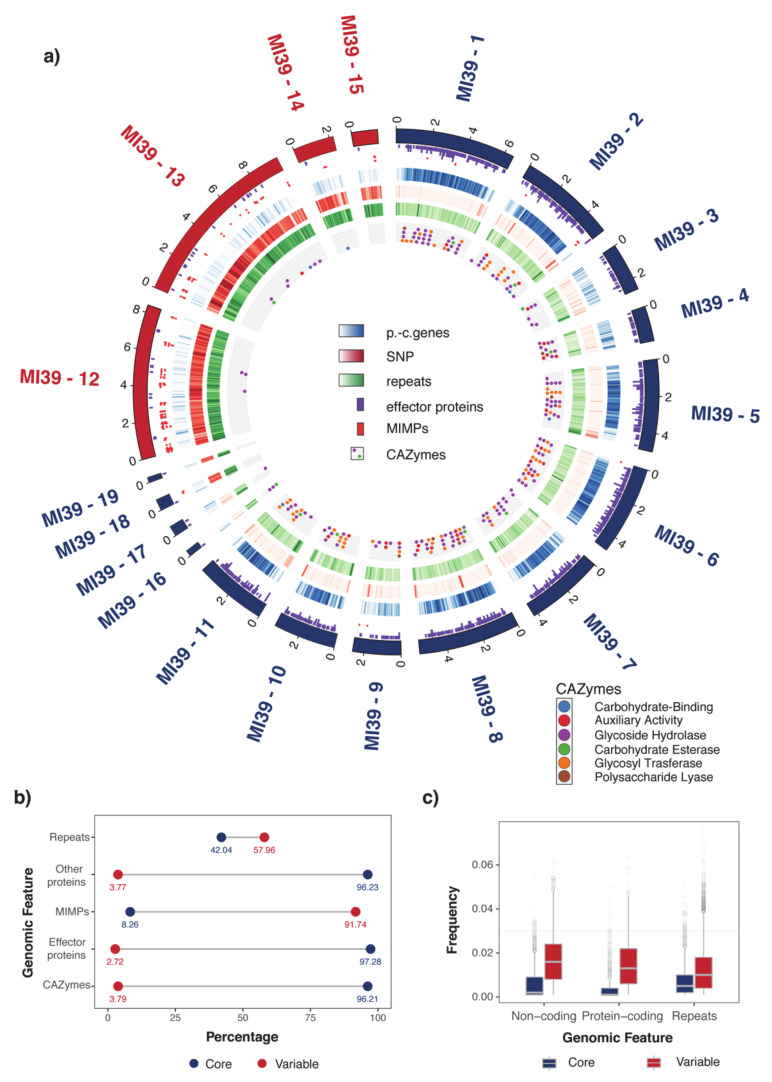
Genomic architecture and properties of *Fusarium oxysporum* f. sp. *lini*. (**a**) Genomic architecture of *Fusarium oxysporum* f. sp. *lini* MI39 isolate visualized with Circos software. The outermost ring depicts chromosome ideogram where chromosomes were assigned to either Core (blue) or Variable (red) compartments of the genome. The next two rings (violet and red ticks) show effector protein loci and MIMPs genomic positions, respectively. Protein-coding genes (p.-c genes), SNP, and repetitive element densities computed in 50 K bins along the chromosomes are presented as blue, red, and green tracks. Color intensity gradient reflects changes in density. The darkest color shade corresponds to maximum density values. The most inner track (grey with circle glyphs) depicts location of CAZymes in MI39 *F. oxysporum* f. sp. *lini* genome. CAZyme types are color-coded as follows: blue—carbohydrate-binding, red—auxillary activity, violet—glycoside hydrolase, green—carbohydrate esterase, orange—glycosyl transferase, brown—polysaccharide lyase. (**b**) Fraction of genomic features associated with either Core (blue) or Variable (red) parts of the genome. Values in a dumbbell chart correspond to percentage of features attributed to either of them. (**c**) Distribution of SNP frequency values computed in various genomic regions (as described in (**a**)) and parts of the genome. Core and variable part is shown in red and blue, correspondingly.

Previously, MI39 whole genome assembly chromosomes were assigned to core and variable (auxiliary, lineage-specific) compartments of the size of 47.55 and 21.77 Mb, correspondingly [20]. Chromosomes were assigned to the core compartment based on high levels of homology and synteny with the core chromosomes of *F. oxysporum* f. sp. *lycopersici*. The synteny-based partitioning is further supported by statistically significant differences in density of protein coding genes, single nucleotide polymorphisms (SNPs), and other genomic features (Figure 1 and Table 1). Thus, in contrast to the variable part, core chromosomes are characterized with much higher density of genes, lower densities of variants, and repetitive regions. In addition, the vast majority of CAZYmes and effector genes are also localized to the core part, whereas mobile DNA (and other repeats including miniature impala repetitive elements (MIMPs) is concentrated in the variable part (Figure 1b and Table 1). The partitioning of the MI39 genome into the core and variable compartments are further supported by significant differences in SNP density (Figure 1c), regardless of its origin e.g., protein-coding, non-coding, and repeats (the associated *p*-values are 2.5 × 10^−225^, 9.5 × 10^−129^, and 5.8 × 10^−110^, correspondingly). 

In contrast to the core part, the lineage-specific chromosomes are characterized by low abundance of genes responsible for pathogenicity and virulence of the fungus, e.g., effectors and CAZYmes (*p*-values 0.002 and 0.02, correspondingly), as shown in Figure 1a, in Figure S1 and Table 1. It has been shown that MIMPs are often found in proximity to genes encoding effector proteins [4]. Moreover, it has been proposed that their proximity could be a predictor for discovery of effector genes [17]. MI39 genome features 230 MIMPs, of which 211 are located in the LS compartment. We did not find statistically significant evidence in support of co-localization of MIMPs and effector genes, as only 11 of them were found within 2 Mb upstream of the nearest effector gene. 

MI39 genome contains 3 SIX gene families, namely SIX1, SIX7, and SIX13 as identified by homology (Figure S1). The majority of them reside on chromosome 12, which contains SIX1 gene, 2 almost identical copies of SIX 13 and 2 copies of SIX7. At the same time, chromosome 15 features an extra copy of SIX7 clearly dissimilar to the rest of the family (Figure S2).

Fusarium produces a diverse spectrum of secondary metabolites of different biosynthetic origin. Genes encoding proteins responsible for synthesis of these metabolites are often clustered on core chromosomes. Using the antiSMASH tool, we predicted 30 secondary metabolite clusters in the MI39 genome, all of which are located in the core compartment. These clusters contain genes encoding non-ribosomal peptide synthase, polyketide synthase, and terpene synthase (Table 1 and Table 2).

Interestingly, a noticeable difference in gene density is observed within core compartment between chromosomes 3, 4, and 9 and others constituents of the core part (*p*-value = 6.8 × 10^−28^) (Figure 1). The aforementioned chromosomes are syntenic to *F. oxysporum* f. sp. *lycopesici* (Foli) chromosomes 11, 12, and 13, which are distinguished from the rest of Fol4287 core chromosomes for different evolutionary properties (gene density, etc.) [4,14]. 

**Figure 2:**
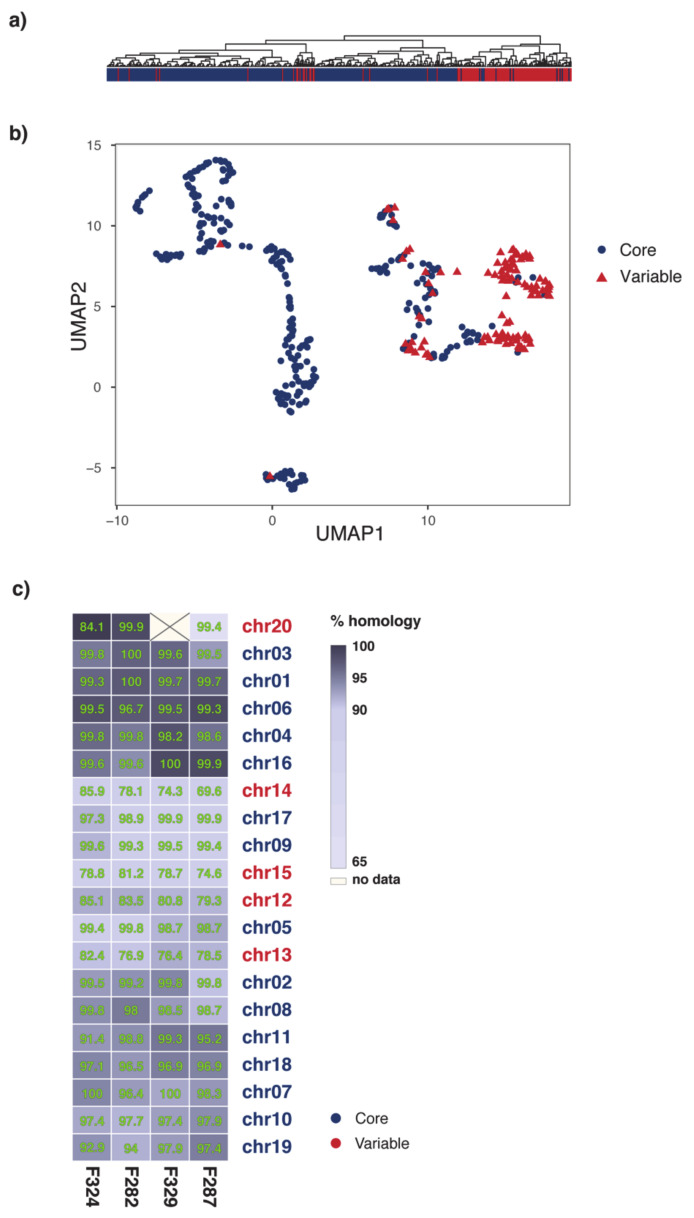
Compartmentalization of *F. oxysporum* f. sp. *lini* genome. (**a**) Hierarchical clustering of genomic regions in accordance with genomic characteristics associated with genome stability. Regions attributed to the Core part are marked with blue, whereas loci from the variable part are shown in red. (**b**) Visualization of genomic region clustering with uniform manifold approximation and projection (UMAP) reveals three groups of loci. (**c**). Homology heatmap for comparisons of the percentage identity between flax strains. The percentage identity is indicated by different box colors, whereas the numbers shown is the boxes correspond to percentage of overlap between chromosomes. Chromosome labels are highlighted according to genome compartmentalization.

An in-depth analysis carried out using various quantitative features related to genome stability and variability hinted at the possibility of non-binary partitioning of FOLINI genome. Clustering of density values computed over SNPs and repeats in 4Kb windows, dN/dS values associated with 2326 protein-coding genes, as well as local genome integrity score (Figure 2a) reveals at least three types of genomic regions. The observed multi-compartmentalization of the genome is further supported by application of uniform manifold approximation and projection (UMAP), a non-linear dimension reduction tool, to the data (Figure 2b). In tune with the above, the extended analysis of chromosome synteny between MI39 and 4 other FOLINI strains shown on a heatmap in Figure 2c, corroborates the evidence for existence of at least three genome partitions.

### 2.2. Functional Annotation of FOLINI Proteome

**Figure 3:**
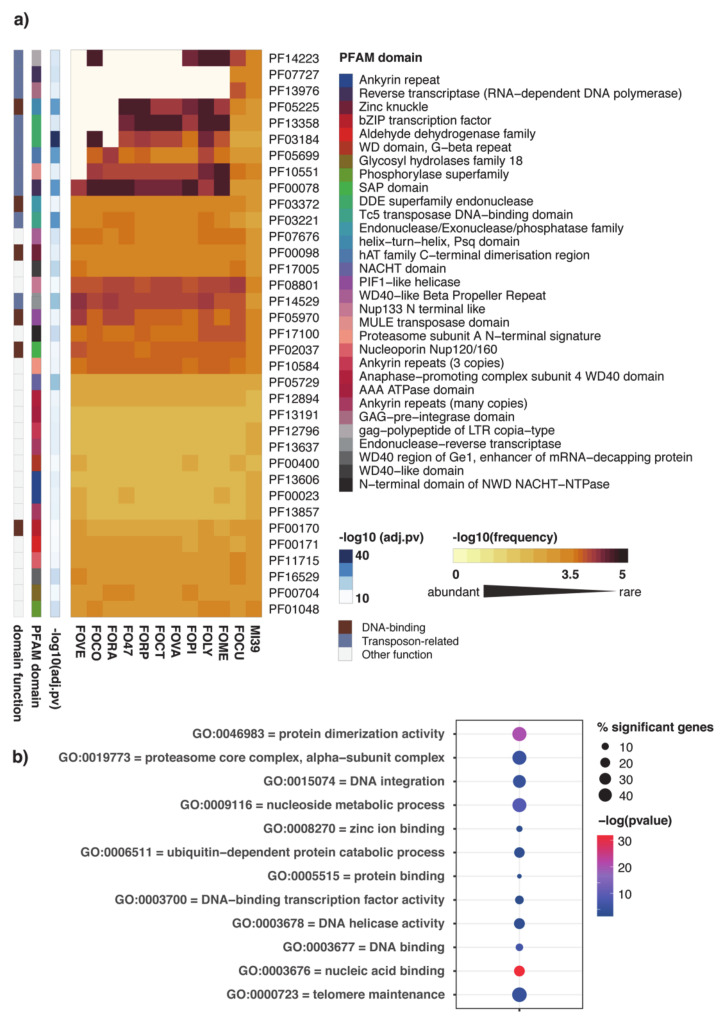
Functional annotation of *F. oxysporum* genomes. Heatmap of PFAM domain frequencies observed in variable part of *F. oxysporum* genomes. **(a)** The heatmap shows 35 PFAM domains significantly overrepresented (adj. *p*-value < 10^−6^) in variable genome of MI39 *F. oxysporum* f. sp. *lini* isolate. Frequency values associated with each domain were mean-centered by rows. Each row of the heatmap represents log10-transformed frequency values of one PFAM domain across all *F. oxysporum* formae speciales (yellow, high frequency; brown, low frequency). Ivory color represents missing data points i.e., situations when a domain has not been detected in a pathogen’s genome. Domain enrichment significance is indicated in shades of blue on a separate panel on the left of the heatmap. The next two panels present domain name for each PFAM accession and information regarding domain function and its relevance to genome mobilization. The abbreviated *F. oxysporum* formae specialis names shown at the bottom of the heatmap stand for: MI39, FOCU—f. sp. *cubense*, FOME—f. sp. *meloni*, FOLY—f. sp. *lycopersici*, FOPI—f. sp. *pisi*, FOVA—f. sp. *vasinfectum*, FOCT—f. sp. *cotton*, FORP—f. sp. *raphani*, FO47—FO47 strain, FORA—f. sp. *radicis*, FOCO—f. sp. *conglutinans*, FOVE—*Fusarium verticilloides*. (**b**) Significantly overrepresented GO terms encompassing processes associated with proteins in the variable compartment of the MI39 genome. Spheres represent GO term enrichment with size and color as indicated in the inset.

A comprehensive genome-wide annotation of FOLINI was performed using the PFAM database, a collection of protein families based on functional domains. PFAM domain frequencies were computed for proteomes of 11 Fusarium species obtained from Ensembl Fungi (i.e., *F. oxysporum* f. sp. *cubense*, *F. oxysporum* f. sp. *meloni*, *F. oxysporum* f. sp. *lycopersici*, *F. oxysporum* f. sp. *pisi*, *F. oxysporum* f. sp. *vasinfectum*, *F. oxysporum* f. sp. *cotton*, *F. oxysporum* f. sp. *raphani*, FO47 strain, *F. oxysporum* f. sp. *radicis*, *F. oxysporum* f. sp. *conglutinans*, *Fusarium verticilloides*) and MI39 proteome. Domain enrichment analysis revealed a group of 35 PFAMs significantly over-represented in LS compartment of MI39. Unsurprisingly, the core part is quite homogeneous in terms of domain repertoire, whereas the LS compartment shows noticeable differences between various *formae specialis* (Figure 3a and Figure S3), thus indirectly underpinning a theory regarding a pivotal role of variable compartment in host adaptation. Interestingly, domain spectrum of MI39 variable proteome is most similar to that of banana pathogen (*F.oxysporum* f. sp. *cubense* race 4, GCA_000350365). Both proteomes are characterized with relatively high frequency of domains associated with activity of transposable elements apparently absent in other f. spp.

To get further insight into biological roles of proteins specific to the variable compartment of MI39, we searched the respective proteome for significantly enriched Gene Ontology (GO) terms (FDR < 0.001). The results encompassing terms such as nucleic acid binding, DNA integration, DNA binding, DNA helicase activity, DNA binding transcription factor activity presented in Figure 3b are suggestive of a toolbox for genome reorganization to potentiate host adaptation processes.

### 2.3. Phylogeny

**Figure 4:**
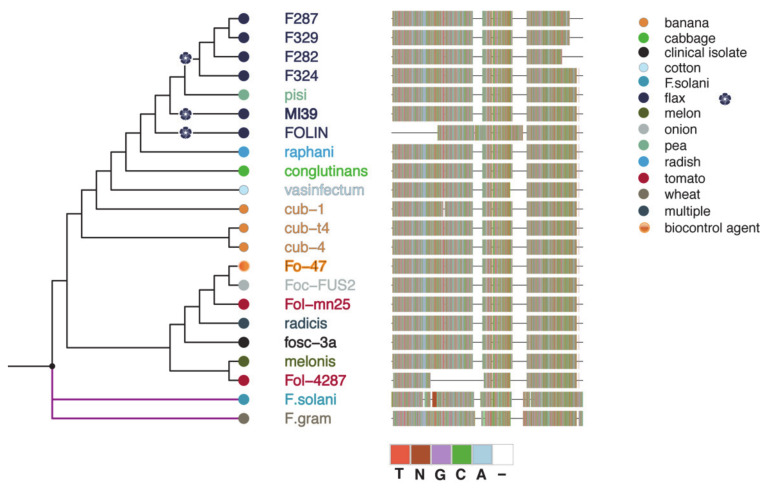
Bayesian phylogeny of *Fusarium oxysporum* isolates from flax and other hosts. The phylogeny is inferred from the maximum likelihood analysis of concatenated alignment of RPB2-TUB2-EF1α and is rooted on the outgroup comprised of *F. solani* and *F. graminearum* branch). Tree tips and label colors reflect the pathogen’s host. The color-coded nucleotide sequence alignments are shown to the right of the tree.

To determine whether the 5 sequenced strains are monophyletic, we inferred RPB2-TUB2-EF1α maximum likelihood phylogeny (Figure 4). The 5 strains of f. sp*. lini* are clustered together with another strain FOLIN of the same *formae specialis* and with strain hdv-247 which belongs to f. sp. *pisi*.

Further in-depth analysis was carried out to confirm whether f. sp. *lini* at all has polyphyletic or monophyletic origin. We tested *F. oxysporum* f. sp. *lini* strains collected from different locations and constructed a phylogenetic tree based on alignment of EF1α sequences (Figure S4). The resulting tree exhibits at least 4 distinct clonal lineages distributed in different *F. oxysporum* clades, confirming that f. sp. *lini* cannot be defined as monophyletic group. This finding is in good agreement with previous phylogenetic analyses of *Fusaria* from flax [21].

**Figure 5:**
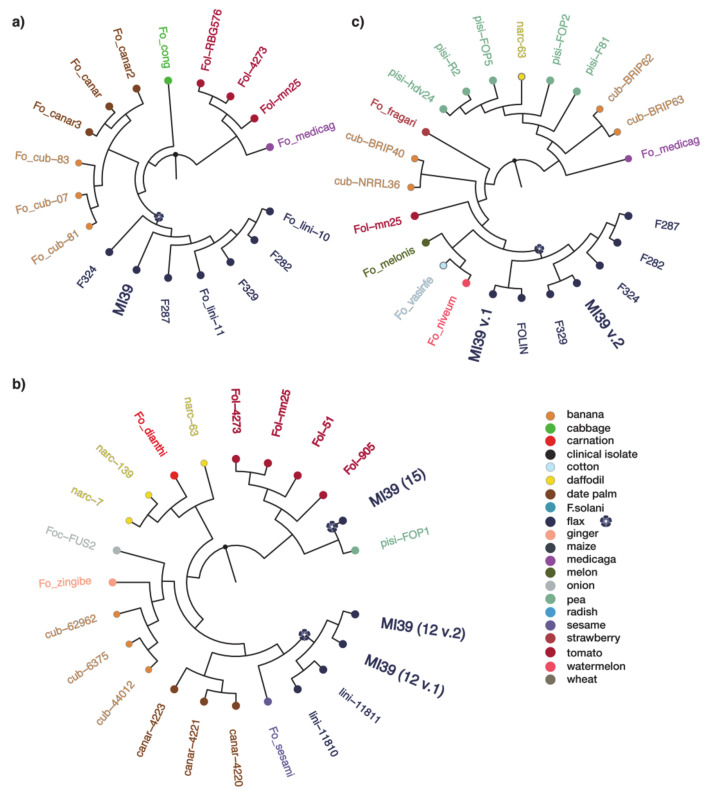
Consensus trees from the Bayesian phylogenetic analysis of SIX genes from the Fusarium clade. Tree tips and label colors reflect the pathogen’s host organism. List of hosts is shown separately. (**a**) SIX1 consensus tree consensus tree, (**b**) SIX7 consensus tree, and (**c**) SIX13 consensus tree.

We also constructed separate phylogenetic trees for SIX1, SIX7, and SIX13 genes to determine their modes of origin (Figure 5). The SIX1 dataset included 18 *F. oxysporum* isolates (Table S1) belonging to six *formae specialis*. SIX1 genes found in f. sp. *lini* cluster together with ff. spp. *cubense* and *canariensis*, and form a well-supported clade with f. sp. *conglutinans*. The cluster is distinct from the clade which includes ff. spp. *lycopersici* and *medicaginis*. Taken together, these observations clearly attest to the hypothesis that f. sp. *lini* SIX1 genes are of monophyletic origin.

The SIX13 gene tree consists of 22 isolates (Table S1) and its monophyletic origin is supported for at least 6 strains of f. sp. *lini* used in this analysis. However, the SIX7 gene phylogeny, consisting of 21 isolates from 10 ff. spp. (Table S1), shows that two copies of this gene located on 12 chromosome form one clade with other f. sp. lini strains, while the third copy on 15 chromosome is phylogenetically distinct and is more related to isolates obtained from *S.lycopersicum* and *P.sativum*. 

We conclude that f. sp. *lini* is of polyphyletic origin as confirmed by housekeeping genes phylogeny. SIX7 gene homologs are of polyphyletic origin as well. In contrast, SIX1 and SIX13 genes detected in the 5 strains of f. sp. *lini* are most likely of single origin as demonstrated in respective phylogenetic trees. 

Multiple sequence alignments of SIX proteins (Figure S2) clearly demonstrate high level of conservation between different strains. Phylogenetic analyses using SIX7 protein sequences show two distinct groups of proteins located on chromosomes 12 and 15. Interestingly, a copy situated on chromosome 15 is quite different from the rest of SIX7 protein sequences analyzed here.

### 2.4. Expression Analysis of Selected Genes within Variable Compartment 

In view of the results of the comparative domain enrichment analysis carried out in 12 FOSC proteomes that demonstrated a significant overrepresentation of domains associated with transposable elements, we hypothesized that expression of proteins harboring those domains could play a significant role in pathogenicity. Specifically, we concentrated on protein domains directly implicated in the life cycle of DNA transposons and retrotransposable elements. Thus, proteins containing gag and RT (reverse transcriptase) domains were obvious candidates for exploratory analyses. From the list of proteins containing the aforementioned domains 10 candidates were selected at random and their expression profiles were assessed in planta. In a like manner, 5 candidates were chosen from the list of proteins containing DDE and endonuclease domains. 

**Figure 6:**
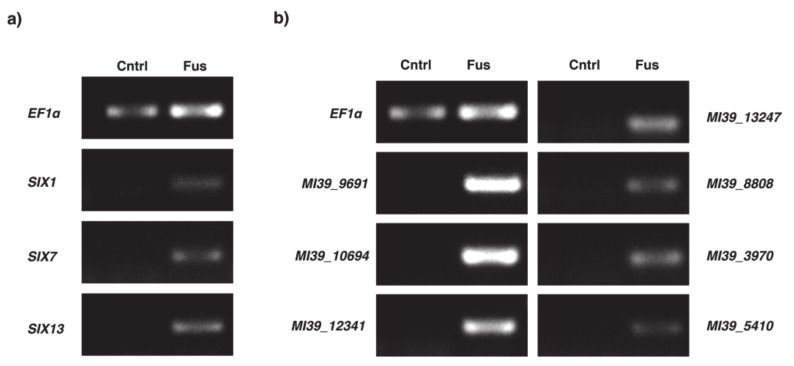
PCR analyses showing the presence of *F.oxysporum* f. sp. *lini* gene products. (**a**) PCR amplification of E1Fα (reference), SIX1, SIX7, and SIX13; (**b**) PCR amplification of genes involved in a life cycle of transposable elements. Control cDNA, extracted from non-infected plant is labeled with Cntrl, whereas Fus marks cDNA obtained from infected flax roots as described in the Materials and Methods section.

To explore their potential role in infection, gene expression levels were assessed with RT-PCR. Furthermore, expression levels of all *SIX* genes identified in FOLINI were profiled as infection markers, whereas *E2F1* and *CYC* were used as reference controls. All 11 genes tested have shown clear expression in planta, suggesting their role in the infection process (Figure 6). 

## 3. Discussion

An extensive genome analysis of 5 strains of *Fusarium oxysporum* f. sp. *lini* revealed a remarkable genome architecture challenging the “two-speed” genome model in the case of this particular pathogen. Stunningly and in sharp contrast to current perception of a “two-speed” genome model, our results show significant differences in numbers of effector proteins, CAZYmes and secondary metabolite clusters found on stable, core chromosomes in vast quantities (Table 1). Evaluation of genomic features, such as densities of SNP, repetitive regions, protein-coding genes etc., commonly used as a proxy for genome plasticity and susceptibility to variation points rather at a feasibility of a “three-speed” or even “multi-speed” evolutionary model, thus questioning the rationale behind over-simplistic theories. Our findings evidence in favor of at least three genomic compartments (Figure 1a and Figure 2), presumably with different evolutionary properties. 

Thus, a compartment distinguished by a relatively low number of genes and repeats, high level of intra-strain synteny, and low level of genetic variation (chromosomes 1, 3, 4, 6, 16) is referred to as a conserved “core” part of the genome. The so-called “variable”, dynamic compartment is just the opposite of the core and includes chromosomes 12–15. This part of the genome is highly syntenic with LS chromosomes of Fol [20] and features low number of protein-coding genes, as well as both high rates of intra-strain variation and density of repeats. Genomic regions comprising rest of the genome (chromosomes 2, 5, 7, 8–11, 17, and 18) exhibit a mixed profile of features which complicate their attribution to either of the compartments. This observation is in tune with recent studies [4,22], suggesting a crosstalk between core and accessory components of the genome.

Pathogen- and virulence-specific genes are not solely bound to the variable genome, hinting at the existence of complex genetic and epigenetic mechanisms in charge of pathogenicity and host adaptation. For instance, two homologs of cerato-plantanin gene FocCP1 [23], responsible for host penetration and virulence in f. sp. *cubense* is located on chromosomes 1 and 5. Conversely, all copies of SIX genes belong to the dynamic genome compartment (chromosomes 12 and 15). Undoubtedly, in-depth analysis involving other omics data modalities is essential to refine the proposed genome compartmentalization scheme to decipher the evolutionary model of fungal pathogenicity.

In contrast to housekeeping genes, members of SIX1 and SIX13 gene families demonstrate host-related clustering as revealed by phylogenetic analyses (Figure 5 and Figure S4), thus being indicative of strong adaptation to host. In this context, SIX7 gene poses a particular interest, as one of its copies is located on different chromosome, is absent from other 4 FOLINI strains sequenced and is more related to SIX7 homologs from f. sp. lycopersici strains, strongly suggesting a relatively recent horizontal transfer event.

Functional analysis of *F. oxysporum* f. spp. protein repertoire reveals the dominating role of the variable compartment in diversification of the *formae specialis*. Remarkably, the main contributors to proteome divergence are PFAM domains associated with DNA-binding and mobilization of transposable elements, possibly suggestive of active transcription and ongoing genome reshuffling in variable compartment (Figure 3). 

Previous research has shown that active transposable elements populate lineage specific compartment of fungal pathogens [24]. It has been hypothesized that variable genome compartment evolves through rearrangements triggered by transposable element activity, which impacts the evolution of pathogen virulence. We were able to observe simultaneous expression of pathogenicity-related genes and genes directly involved in a life cycle of both DNA transposons and retroelements. This may support the aforementioned hypothesis that evolution of *Fusarium* genomes and remarkable adaptability of the fungus to the host are for the most part shaped and driven by the activity of mobile elements. Contrarily, the abundant expression of transposable elements RNA could be a consequence of extensive chromatin re-modeling concomitant to the infection process. Thus, further insight into chromatin biology of *F. oxysporum* f. sp. *lini* will be instrumental to enhance our perception of the evolution of its multi-compartmentalized genome and on its pathogenicity.

Parasitic genome is arguably a center-point of adaptation destined to counterweight the host immune system and environmental challenges. A new genomic blueprint of a fungal pathogen lays a solid foundation for in-depth studies of molecular mechanisms of species divergence, adaptation, and pathogenicity, thus boosting our efforts to fight and control fungal infections. 

## 4. Materials and Methods

### 4.1. Data Sources and Sequence Analysis

The nucleotide sequences assembly, de novo prediction, and annotation of coding regions of monoisolate 39 and 4 strains (282, 289, 324, 327) have been described in our previous publication [20]. De novo prediction of repetitive sequences was made using RepeatScout [25] version 1.0.6 MIMPs have been predicted using custom Perl scripts and regular expressions/CAGTGGG.GCAA[TA] AA/and/TT[TA]TTGC.CCCACTG/, described in [4]. Short reads of 4 FO strains have been aligned to MI39 reference genome using bwa-mem [26] with default parameters. Variant calling for the alignments have been done using NGSEP [27] version 4.0. Synteny analysis was done using Satsuma2 program [28]. Genome stability metrics was computed as a local aberration score with spector R package (https://github.com/anasrana/spector, accessed 1 December 2020).

### 4.2. Proteins Annotation

Protein sequences predicted by BUSCO [29] as described earlier (Table S3). Secondary metabolite clusters analysis was done using Fungal antismash server version 5.1.2 [30] with the following types of analysis: KnownClusterBlast, ClusterBlast, SubClusterBlast, ActiveSiteFinder, Cluster Pfam analysis, and Pfam-based GO term annotation. CAZYme proteins have been predicted with dbCAN meta server [31] using HMMER, DIAMOND, and Hotpep prediction methods. Effector proteins prediction was made using EffectorP package version 2.0 [32]. Search for PFAM domains in the protein sequences was done for PFAM database version 33.1 using HMMER version 3.3 [33]. Search for orthologous groups of proteins was made with orthoMCL version 2.0.9 [34]. Orthology analysis identified genes common between Foli strains and 16 other proteomes from FOC, 97.1% proteins were clustered into 23,975 orthogroups, 7239 orthogroups were shared between all 16 proteomes and represented 187,648 proteins, while 3065 orthogroups contained proteins in a 1:1 relationship between MI39 proteome and others FOSC.

### 4.3. Phylogenetic Analysis

For the phylogenetic analysis of housekeeping genes, we used publicly available sequences of the genes on NCBI: RPB2 (GB: KX434985.1), EF1α (GB: KC889026.1), and TUB2 (GB: KP964895.1) as queries for BLASTN search. Databases were created using makeblastdb script for our published genomes of *Fo* f. sp. *lini*, 12 genomes from Ensembl Fungi (Table S1), *Fo* f. sp. *lycopersici* Fol-4287, and *Fo* f. sp. *cepae* Foc-FUS2 genomes from NCBI. BLASTN search was performed with default parameters. Sequences with identity more than 90% (1 sequence for each of 3 genes of each species) were extracted from BLAST databases using blastdbcmd script. Sequences of the same genes of *Fusarium graminearum* and *F. solani* were downloaded from NCBI and used as outgroup. Each of 3 gene datasets were aligned using ClustalX [35] and then 3 resulting alignments were concatenated together with MEGA X software [36]. Maximum likelihood phylogeny was inferred in MEGA X by using Tamura-Nei model [37] with discrete Gamma distribution (4 categories, G-parameter = 1.0613) and 1000 bootstrap replicates. The phylogenetic trees were visualized in FigTree and rooted on the outgroup. The resulting trees are presented as cladograms (see Figure 4, Figure 5 and Figure S4), for clarity of presentation reasons only.

For the phylogenetic analysis of EF1α gene sequences were selected as described above. We also added 15 sequences of EF1α from other strains of f. sp. *lini* available on NCBI and one strain XJ37 belonging to f. sp. *cubense* (Table S2). Sequences of EF1α from *F. graminearum*, *F. verticelloides*, and *F. solani* from NCBI were used as outgroup. Then we aligned 37 sequences using ClustalX and manually curated the alignment. Maximum likelihood phylogeny was estimated and tree was constructed with IQ-TREE software [38]. We used Tamura-Nei + G4 substitution model and 1000 bootstrap replicates. Resulting tree was curated with FigTree as described above. The phylogenetic analysis of SIX1, SIX7, and SIX13 genes was carried out using available sequences of these genes for different FOSC ff. spp. obtained either from NCBI or from Ensembl Fungi (see Table S2). To infer tree structure for each gene, we performed a multiple sequence alignment with ClustalX and subsequent phylogeny analysis with IQ-TREE. Substitution models were chosen in accordance with ModelTest as implemented in IQ-TREE software. Next, the consensus trees from 1000 bootstrap replicates were built. The resulting trees were midpoint rooted and curated using FigTree. The phylogeny of protein sequences of SIX genes products was inferred from multiple sequence alignments as implemented in MAFFT software (L-INS-i algorithm, 1000 iterations). Trees visualization was created with ggtree R package [39].

### 4.4. RNA Isolation, Reverse Transcript, and PCR

For gene expression analysis, flax roots after inoculation with MI39 isolate for 3 days were sampled. As a control, we used non-infected plants. The root was washed clean, cut from the plants, and used for RNA extraction with RNeasy Mini Kit (QIAGEN, Germany) as per manufacturer’s instructions. After quantification using NanoDrop 8000, the first strand of cDNA was synthesized with MMLV-RT commercial kit (Evrogen, Russia). We used Oligo(dT)17-primer for first strand cDNA synthesis, as per the manufacturer’s instructions. The PCR was carried out using the Veriti 9000 instrument (Applied Biosystems) in technical triplicate using the indicated primer pairs (see Table S4). Agarose gel electrophoresis was used to separate the amplified fragments of cDNA. We used Quantum-ST gel-documenting system and software (Vilber Lourmat) to visualize the results of PCR and curate images.

## Figures and Tables

**Table 1:** Characteristics of *F. oxysporum* f. sp. *lini* monoisolate 39 reference genome. Densities of genomic elements per 1 Mb and *p*-values in support of significant difference in the densities between core and variable parts of the genome.

		Genomic Elements							
Chromosome	Length (Mb)	Proteins	MIMPs	Secondary Metabolites Clusters	CAZYmes	Effector Genes	Repeats	SNPs	TFs
MI39-1	6.5	372.93	0.615	0.307	6.76	36.00	263.39	186.00	3.076
MI39-2	5.28	343.92	1.704	0.946	5.87	29.54	254.34	505.65	2.651
MI39-3	2.64	93.33	0	0.379	2.65	9.86	257.61	335.01	0
MI39-4	1.9	149.64	0	0	8.95	18.96	186.53	245.01	0
MI39-5	4.99	315.10	0	0.400	7.80	34.61	279.09	328.50	0.601
MI39-6	4.82	389.17	0.414	0.622	8.50	40.22	244.24	101.59	2.489
MI39-7	4.23	301.70	0	0.472	4.24	27.38	269.36	147.78	2.364
MI39-8	5.51	316.39	0	0.181	8.89	31.23	221.22	475.86	1.633
MI39-9	2.62	101.66	0.764	0.382	3.82	13.37	218.22	491.87	0
MI39-10	3.36	273.75	0	0	5.93	25.23	295.12	404.39	1.785
MI39-11	3.9	312.49	0	0	6.40	28.94	323.51	404.71	1.282
MI39-12	8.4	19.86	17.84	0	0.35	1.784	830.75	9296.25	0.238
MI39-13	9.7	23.18	4.84	0	0.82	1.957	828.96	9924.00	0.103
MI39-14	2.21	35.66	0.902	0	0.45	0.902	803.50	6367.08	0
MI39-15	1.41	19.81	8.49	0	0	0.707	834.91	5314.45	0.709
MI39-16	0.33	45.33	0	0	48.35	9.06	507.72	456.34	0
MI39-17	0.56	51.52	0	0	30.20	10.66	483.25	577.41	0
MI39-18	0.61	29.48	3.27	1.637	0	1.637	804.19	5929.12	0
MI39-19	0.28	25.34	0	0	0	3.620	1035.39	7247.74	0
MI39-20	0.035	0	0	0	0	0	688.17	200.71	0
*p*-value (core/variable)		0.002	0.003	0.058	0.024	0.002	0.009	0.003	0.677

**Table 2:** Secondary metabolites clusters.

Gene ID	Chromosome	Start	End	Type	Most Similar Known Cluster	Similarity
MI39_11795	MI39-1	396,772	398,794	NRPS		
MI39_7258	MI39-1	592,293	593,294	saccharide		
MI39_7862	MI39-1	967,185	970,941	NRPS-like		
MI39_1628	MI39-1	2,347,825	2,349,019	saccharide		
MI39_10036	MI39-2	298,534	305,960	T1PKS		
MI39_231	MI39-2	2,684,091	2,688,260	NRPS-like		
MI39_13258	MI39-2	2,684,091	2,688,694	NRPS-like		
MI39_2278	MI39-2	3,261,368	3,262,567	saccharide		
MI39_14038	MI39-2	3,765,374	3,773,009	fatty_acid		
MI39_2573	MI39-2	3,765,377	3,768,012	fatty_acid		
MI39_9444	MI39-2	3,765,849	3,768,012	fatty_acid		
MI39_12401	MI39-2	4,574,044	4,577,898	NRPS-like		
MI39_11789	MI39-2	4,575,793	4,577,898	NRPS-like		
MI39_1311	MI39-3	1,671,638	1,674,973	NRPS-like		
MI39_9164	MI39-5	232,718	235,765	NRPS-like		
MI39_1436	MI39-5	2,035,026	2,036,282	terpene		
MI39_10721	MI39-6	699,152	703,444	T1PKS	naphthopyrone	Polyketide 100%
MI39_2845	MI39-6	1,385,216	1,386,634	terpene		
MI39_12234	MI39-6	1,385,216	1,386,765	terpene		
MI39_7678	MI39-6	2,432,577	2,438,251	fatty_acid		
MI39_10493	MI39-6	2,432,577	2,435,482	fatty_acid		
MI39_10754	MI39-6	2,432,577	2,436,631	fatty_acid		
MI39_13479	MI39-7	383,162	391,130	T1PKS		
MI39_5620	MI39-7	2,604,581	2,606,040	T3PKS		
MI39_8099	MI39-8	3,062,963	3,066,332	NRPS-like		
MI39_5810	MI39-9	1,725,235	1,737,100	T1PKS, NRPS	ACT-Toxin II	Polyketide 100%
MI39_602	MI39-10	1,433,101	1,435,614	saccharide		
MI39_9909	MI39-10	1,433,101	1,434,782	saccharide		
MI39_4863	MI39-11	2,099,210	2,100,758	saccharide		
MI39_8184	MI39-18	373,737	383,484	T1PKS, NRPS		

## Data Availability

The data that support the findings of this study are openly available in NCBI GenBank at https://www.ncbi.nlm.nih.gov/genbank/, reference numbers: JABJUA000000000, JABJUB000000000, JABJUC000000000, JABJUD000000000, JABJUE000000000, accessed 03.09.2020.

## Supplementary Materials

The following are available online at https://www.mdpi.com/1422-0067/22/5/2665/s1, Figure S1: MI39 *F. oxysporim* f.sp. *lini* variable chromosomes, Figure S2: Multiple sequence alignments and phylogeny of SIX proteins, Figure S3: PFAM domain repertorie in the core part of *F.oxysporum* genomes, Figure S4: Phylogeny of *Fusarium oxysporum* isolates based on alignment of EF1α gene sequences, Table S1: Identifiers of SIX genes sequences in public databases, Table S2: Identifiers of EF1α genes sequences in public databases, Table S3: Annotation of predicted protein coding genes in a genome of MI39 monoisolate of *F. oxysporum* f.sp. *lini*, Table S4: Primer sequences used in PCR experiments.
