# Supplementary material for: A Genomic Blueprint of Flax Fungal Parasite Fusarium oxysporum f. sp. lini
Source: Int J Mol Sci. 2021 Mar 6;22(5):2665. doi: 10.3390/ijms22052665 (PMC7961770; doi:10.3390/ijms22052665)
Supplement: Supplementary file 1 [file ijms-22-02665-s001.zip › ijms-1134917-supplementary/supplemental_info/SF1.docx]

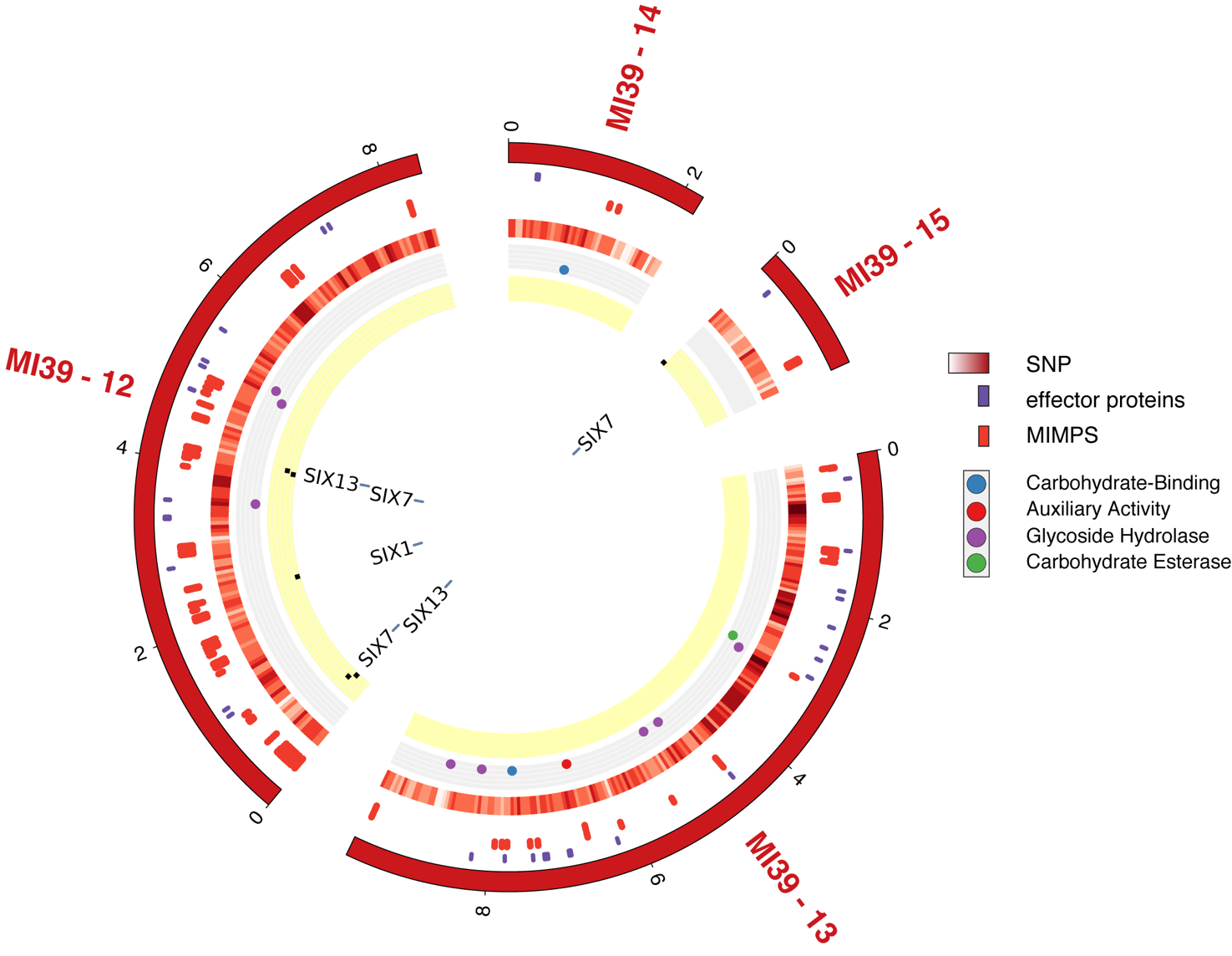


Supplemental Figure 1. **MI39 F. oxysporim f.sp. lini variable chromosomes**

Circos diagram of the MI39 F. oxysporim f.sp. lini variable genome compartment. The outermost ring depicts chromosome ideogram. The next two rings (violet and red ticks) show effector protein loci and MIMPs genomic positions, respectively. SNP densities computed in 50K bins along the chromosomes are presented as red heatmap tracks where colour intensity gradient reflects changes in density. The darkest color shade corresponds to maximum density values. Circle glyphs on grey background depict location of CAZymes. SIX gene locations are labelled separately and are shown as black squares on the most inner yellow track.
