# Supplementary material for: A Genomic Blueprint of Flax Fungal Parasite Fusarium oxysporum f. sp. lini
Source: Int J Mol Sci. 2021 Mar 6;22(5):2665. doi: 10.3390/ijms22052665 (PMC7961770; doi:10.3390/ijms22052665)
Supplement: Supplementary file 1 [file ijms-22-02665-s001.zip › ijms-1134917-supplementary/supplemental_info/SF2.docx]

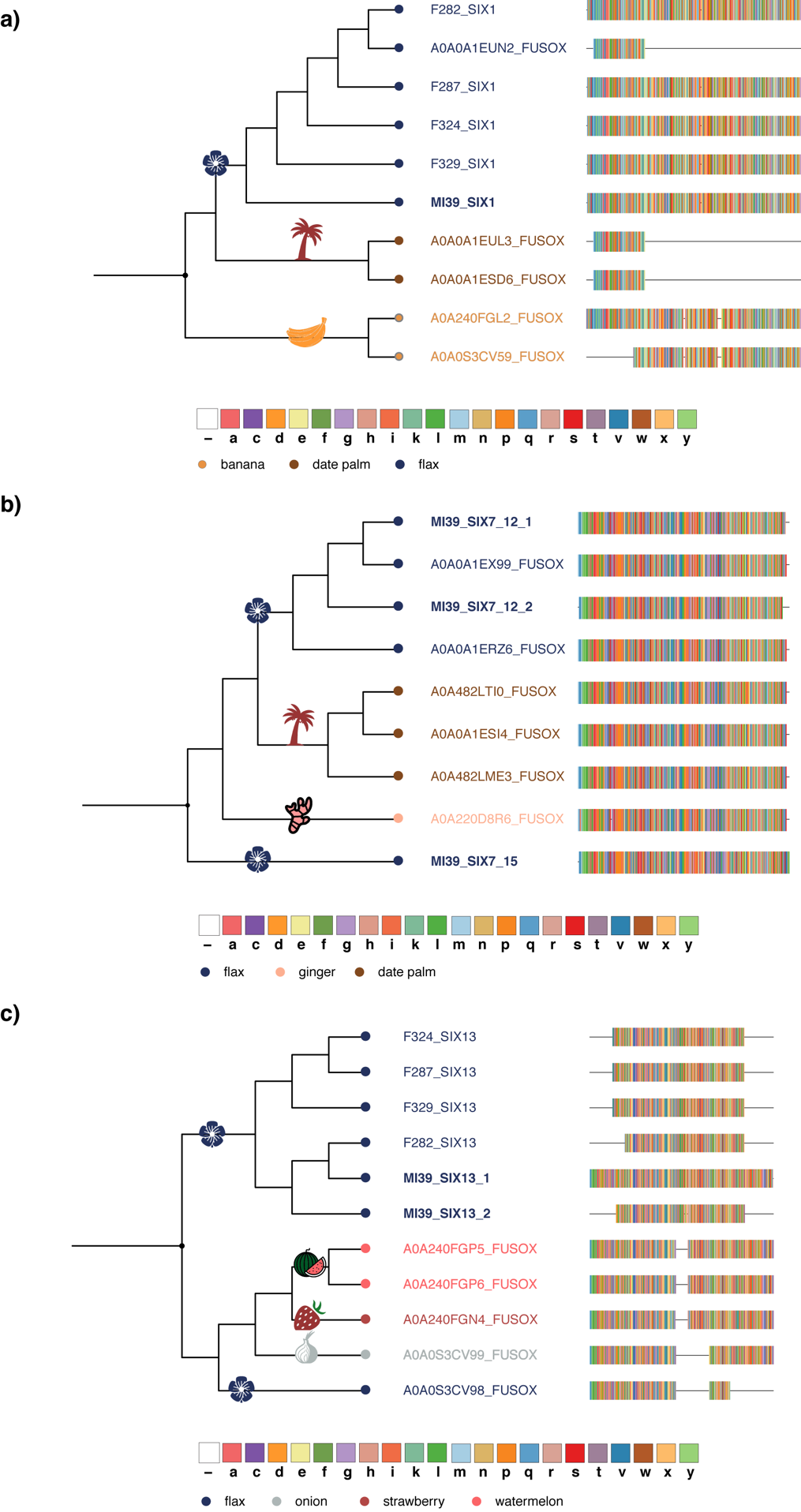


Supplemental Figure 2. **Multiple sequence alignments and phylogeny of SIX proteins.**

The phylogeny is inferred from multiple sequence alignments as implemented in MAFFT software (L-INS-i algorithm, 1000 iterations). Tree tips and label colors reflect the pathogen’s host. Hosts are outlined below each tree. The color-coded amino acid sequence alignments shown to the right of the tree. Icons of the hosts/substrates are drawn on top of the consensus tree branches. All variants of protein sequences as identified by BLAST are included. a) SIX1, b) SIX7, c) SIX13.
