# Supplementary material for: A Genomic Blueprint of Flax Fungal Parasite Fusarium oxysporum f. sp. lini
Source: Int J Mol Sci. 2021 Mar 6;22(5):2665. doi: 10.3390/ijms22052665 (PMC7961770; doi:10.3390/ijms22052665)
Supplement: Supplementary file 1 [file ijms-22-02665-s001.zip › ijms-1134917-supplementary/supplemental_info/SF3.docx]

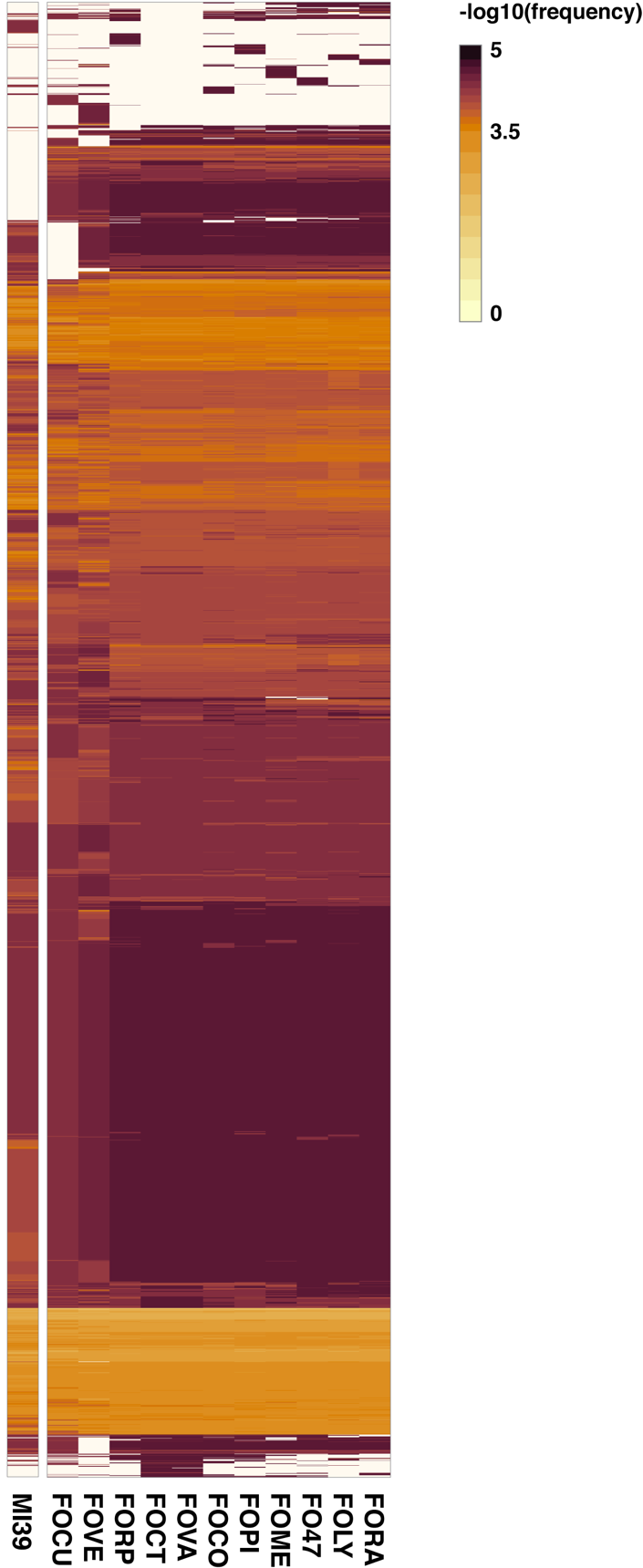


Supplemental Figure 3. **PFAM domain repertorie in the core part of F.oxysporum genomes.**

The heatmap shows PFAM domains repertorie in the core part of MI39 isolate genome and genomes of other ff. spp. Frequency values associated with each domain were mean-centered by rows. Each row of the heatmap represents log10-transformed frequency values of one PFAM domain across all F. oxysporum formae speciales (yellow, high frequency; brown, low frequency). Ivory color represents missing data points i.e. situations when a domain has not been detected in a pathogen’s genome.
