# Supplementary material for: A Genomic Blueprint of Flax Fungal Parasite Fusarium oxysporum f. sp. lini
Source: Int J Mol Sci. 2021 Mar 6;22(5):2665. doi: 10.3390/ijms22052665 (PMC7961770; doi:10.3390/ijms22052665)
Supplement: Supplementary file 1 [file ijms-22-02665-s001.zip › ijms-1134917-supplementary/supplemental_info/SF4.docx]

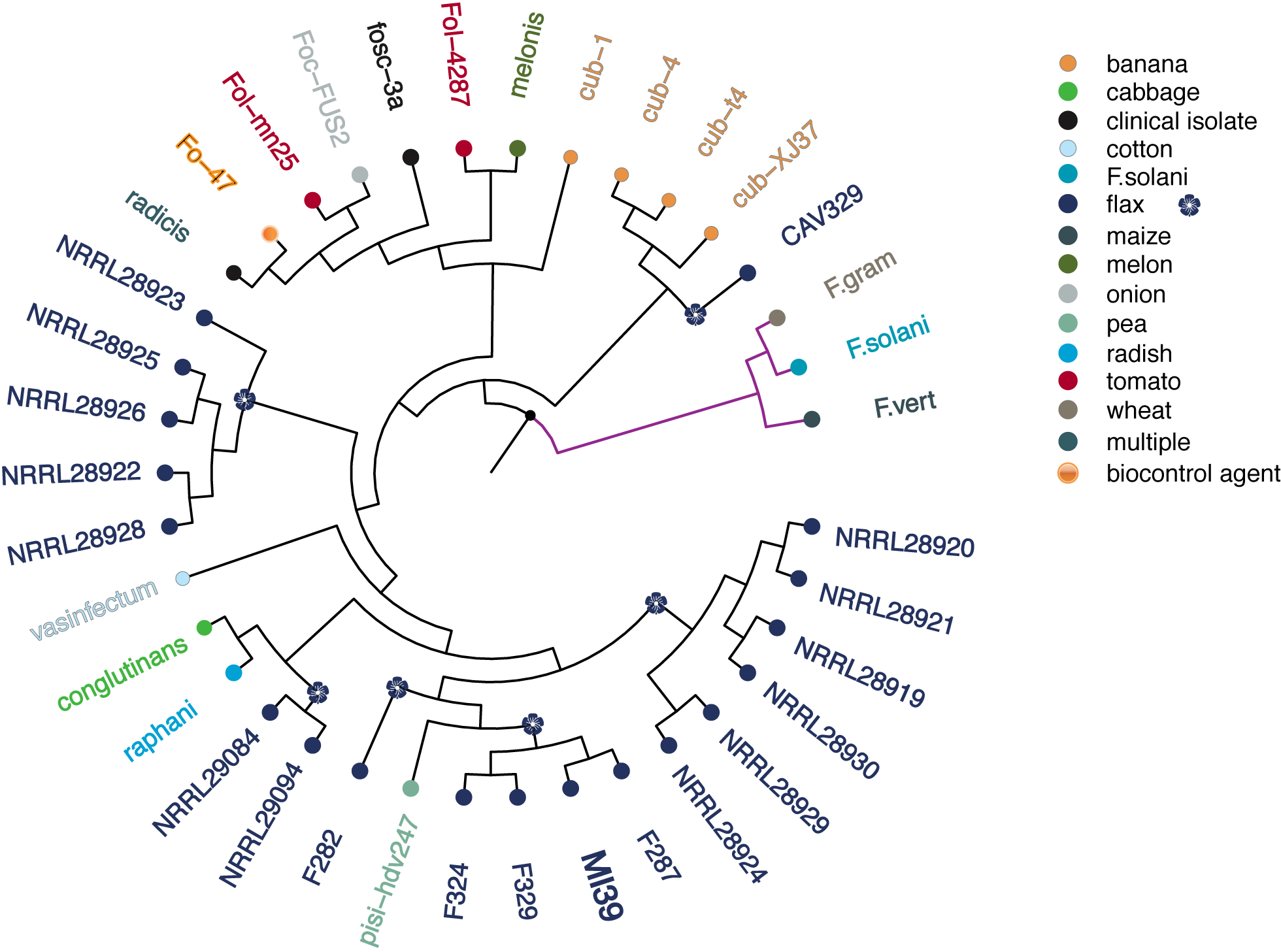


**Supplemental Figure 4. Phylogeny of Fusarium oxysporum isolates based on alignment of EF1α gene sequences.**
Bayesian phylogeny of Fusarium oxysporum isolates based on alignment of EF1α gene sequences from different f. sp. strains within the Fusarium genus (Supplemental Table 1), rooted on the outgroup comprised of F. solani, F. graminearum and F. verticelloides (violet branch). Tree tips and label colors reflect the pathogen’s host.
