# Supplementary material for: A Genomic Blueprint of Flax Fungal Parasite Fusarium oxysporum f. sp. lini
Source: Int J Mol Sci. 2021 Mar 6;22(5):2665. doi: 10.3390/ijms22052665 (PMC7961770; doi:10.3390/ijms22052665)
Supplement: Supplementary file 1 [file ijms-22-02665-s001.zip › ijms-1134917-supplementary/supplemental_info/ST4.docx]

Tables S4: Primer sequences used in PCR experiments

| Name | Gene | Sequence | Rating (Beacon Designer 7.9) |
| --- | --- | --- | --- |
| 8808 | MI39_8808 | 5’-CACATCCACAACAGAG-3’  5’-ACAGCCGACTCACATAGA-3’ | 90,8  91,1 |
| 3970 | MI39_3970 | 5’-AGCGAACTGAGACTCCAATGT-3’  5’-CCTCGGTGTGCTGAAGATAATG-3’ | 88,4  91,1 |
| 5410 | MI39_5410 | 5’-TGTTGATGAGACGCCTATAACG-3’  5’-ACTTCTGATAGTCTTCACCGAGAT-3’ | 86,5  81,3 |
| SIX-1 | SIX-1 | 5’-CGCCCTCTCAATCCT-3’  5’-GAACCGCAGCCTCTT-3’ | 86,7  85,7 |
| SIX-7 | SIX-7 | 5’-ACTTCGGTCTGCCTTCATACT-3’  5’-TTCATTAAGCGGTTGGCGTATT-3’ | 91,1  89,7 |
| SIX-13 | SIX-13 | 5’-TCTCCTGGTTCTCCTATTGCTTA-3’  5’-CCGTTCCGTATGCTCTGTTAG-3’ | 90,7  90,8 |
| *EF1a | EF1a | 5'-GCTGCCAACTTCACATCTCA-3'  5'-GATCGCCTGTCAATCTTGGT-3' | – |
| *CYC | CYC | 5'-TGATTGCGGTCAGCTGTAAG-3'  5'-AGGTGAAACGCTAGGCAGAA-3' | – |
| 10694 | MI39_10694 | 5’-AACATACGCAAGCACTATACGA-3’  5’-GGTCCACATCCACATTCACA-3’ | 88,6  88,0 |
| 12341 | MI39_12341 | 5’-TGTGAGAGCGAGTATCCAGAA-3’  5’-GACCAATATGCCGATGCGATA-3’ | 90,7 90,1 |
| MI39_10205 | MI39_10205 | 5’-TGTGAGAGCGAGTATCCAGAA-3’  5’-GACCAATATGCCGATGCGATA-3’ | 90,7  90,1 |
| MI39_13274 | MI39_13274 | 5’-GATACTGCGTCGGCTCTG-3’  5’-CTTGATGCGGATGAACTGGTA-3’ | 88,8  89,9 |
| MI39_3799 | MI39_3799 | 5’-CACCAGTCCAAGTCGTAGAGA-3’  5’-AGGTTGTAGGCTGCTGAAGA-3’ | 91,1  91,1 |
| MI39_4156 | MI39_4156 | 5’-AACTGCTCCTGCCACTACATA-3’  5’-CCGTCTGCTACCTGCTCATA-3’ | 91,1  90,9 |
| MI39_6166 | MI39_6166 | 5’-CTATCTGCCGCCTCTATCCAT-3’  5’-CTCCGCTCCGCTATCTGT-3’ | 91,0  91,1 |
| MI39_5154 | MI39_5154 | 5’-CCAGTCCAAGTCGTAGAGAAGT-3’  5’-AGGTTGTAGGCTGCTGAAGA-3’ | 90,8  91,1 |
| MI39_1222 | MI39_1222 | 5’-AGCAGAGCAAGAGGAGATGA-3’  5’-GTGTTCCAGCCAGCAGAG-3’ | 90,8  90,3 |
| MI39_4909 | MI39_4909 | 5’-GAAGAGGAAGTTGGCGAATCAT-3’  5’-CGTGGAAGGCATCATTGGAA-3’ | 91,1  91,1 |
| MI39_8813 | MI39_8813 | 5’-CCGTCGCCGCAGATAAGTA-3’  5’-GGTAGCCAGCAGCATCAGA-3’ | 88,7  88,6 |
| MI39_9691 | MI39_9691 | 5’-GAGGCTCAACAACAGACAGTAG-3’  5’-GATTCCACCACGACAGACTC-3’ | 89,2  89,0 |
| MI39_6241 | MI39_6241 | 5’-GTCTACTGATGCCGCTCTC-3’  5’-GCTCGTCGTTATGGTGGAA-3’ | 87,7  88,4 |

*From Huis et al. BMC Plant Biology 2010, 10:71

Huis, R., Hawkins, S. & Neutelings, G. Selection of reference genes for quantitative gene expression normalization in flax (Linum usitatissimum L.). BMC Plant Biol 10, 71 (2010). https://doi.org/10.1186/1471-2229-10-71
